# Engineered Polymersomes for the Treatment of Fish Odor Syndrome: A First Randomized Double Blind Olfactory Study

**DOI:** 10.1002/advs.201903697

**Published:** 2020-03-09

**Authors:** Aaron C. Schmidt, Erik R. Hebels, Charlotte Weitzel, Anna Kletzmayr, Yinyin Bao, Christian Steuer, Jean‐Christophe Leroux

**Affiliations:** ^1^ Institute of Pharmaceutical Sciences Department of Chemistry and Applied Biosciences ETH Zurich 8093 Zurich Switzerland

**Keywords:** biodetoxification, engineered polymersomes, fish odor syndrome, trimethylamine, trimethylaminuria

## Abstract

Trimethylamine (TMA) is a metabolite overtly present in patients suffering from trimethylaminuria (TMAU), a rare genetic disorder characterized by a strong “fishy” body odor. To date, no approved pharmacological treatment to sequester excess TMA on the skin of patients exists. Here, transmembrane pH gradient poly(isoprene)‐*block*‐poly(ethylene glycol) (PI‐*b*‐PEG) polymersomes are investigated for the topical removal of TMA. PI‐*b*‐PEG amphiphiles of varying chain length are synthesized and evaluated for their ability to form vesicular structures in aqueous media. The optimization of the PI/PEG ratio of transmembrane pH gradient polymersomes allows for the rapid and efficient capture of TMA both in solution and after incorporation into a topical hydrogel matrix at the pH of the skin. A subsequent double blind olfactory study reveals a significant decrease in perceived odor intensity after application of the polymersome‐based formulation on artificial skin substrates that has been incubated in TMA‐containing medium. This simple and novel approach has the potential to ease the burden of people suffering from TMAU.

## Graphical abstract

Fish odor syndrome is a metabolic disorder, related to the excretion of high amounts of the foul smelling odorant trimethylamine (TMA). A topical formulation, based on pH gradient poly(isoprene)‐*b*‐poly(ethylene glycol) polymersomes, readily captures TMA in solution. Furthermore, it is capable of lowering perceived odor intensity, when being applied onto TMA‐containing skin surrogates.

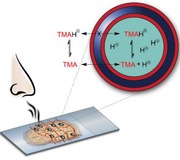

Trimethylaminuria (TMAU), also known as the “fish odor syndrome,” is a genetic disorder related to the excretion of elevated levels of the foul‐smelling odorant trimethylamine (TMA), making sufferers secrete an odor resembling that of rotten fish. While hundreds of cases have been reported in literature in the past decades, the number of affected individuals is largely unknown since this condition is considered widely undiagnosed.^[^ ^1^, ^2^ ^]^ Incidence rates of heterozygous carriers are suggested to be in the order of 1% in the white British population, and are assumed to be higher in other ethnic groups.^[^ ^3^ ^]^

The smell of TMA, a highly volatile tertiary amine, is readily detectable by humans at levels as low as the µg L^−1^ range.^[^ ^4^ ^]^ It is primarily produced by the intestinal breakdown of dietary precursors by colonic bacteria, the vast majority being choline, carnitine, and TMA N‐oxide (TMAO).^[^ ^5^ ^]^ After entering systemic circulation, TMA is oxidized in the liver by the enzyme flavin‐containing monooxygenase 3 (FMO3) to the nonodorous N‐oxide, which is subsequently excreted in the urine. TMAU is caused by an inherited deficiency secondary to mutations in this enzyme, yielding a dysfunctional metabolism of TMA. So far more than 30 sequence variants of the FMO3 gene have been reported to cause the disease.^[^ ^6^ ^]^ Deficient oxidation of the metabolite results in increased systemic levels of TMA, which is excreted through sweat, breath, urine, and other bodily secretions, resulting in an offensive fish‐like body odor.

Although the condition appears to be of little medical concern, it can be devastating from a psychosocial perspective. Literature refers to a range of psychological responses, including signs of mental depression and even suicidal tendencies in some cases.^[^ ^7^, ^8^ ^]^ Currently there is no treatment for TMAU, but only preventive measures, such as dietary restrictions of TMA precursors and frequent washing with acidic soap.^[^ ^9^, ^10^ ^]^ Dietary restrictions can be problematic especially since choline is vital in the formation of essential membrane phospholipids in humans.^[^ ^11^ ^]^ There are various reports studying the use of antibiotics to deplete the microorganisms responsible for TMA generation in the large intestine, but most are inconclusive and only refer to small numbers of patients.^[^ ^12^, ^13^ ^]^ In addition, chronic antibiotic therapy might have a negative impact on the patients' gut microbiota and contribute to antibiotic resistance.^[^ ^14^, ^15^ ^]^ To address this unmet medical need and improve the patients' quality of life, the development of novel treatment approaches is of high urgency.

One approach, that has so far not been tested for the treatment of TMAU, encompasses the sequestration of TMA into vesicular structures. The low molecular weight and basic character (p*K*_a_ = 9.80)^[^ ^16^ ^]^ of TMA make this metabolite suitable for uptake into transmembrane pH gradient vesicles (**Figure** **1**a). The underlying principle is the rapid diffusion of the nonprotonated amine species across the membrane and its subsequent protonation inside an acidic core, trapping it within the vesicle lumen. Our group has previously shown that liposomal carriers bearing a pH gradient could efficiently^[^ ^17^ ^]^ and in a relatively selective fashion^[^ ^18^ ^]^ capture the smaller metabolite ammonia in the peritoneal space. This system lowered systemic ammonia and brain edema when applied via peritoneal dialysis in bile‐duct ligated rats, a model of hyperammonemia.^[^ ^19^ ^]^ However, in more hostile environments, such as the intestine, liposomal formulations can be readily destabilized and release their content.^[^ ^20^ ^]^

**Figure 1:**
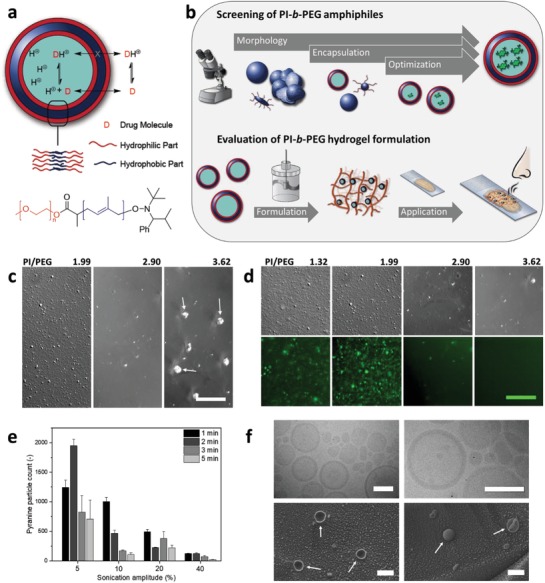
Capture of trimethylamine (TMA) via transmembrane pH gradient polymersomes (top), chemical structure of PI‐*b*‐PEG (bottom) (a). Outline of the screening process of PI‐*b*‐PEG polymersomes by light microscopy (top) and of the subsequent in human olfactory testing with PI‐*b*‐PEG polymersome hydrogel formulation (bottom) (b). Representative images of different morphological data recorded with a light microscope (DIC channel, scale bar 50 µm) (c). Fluorescence microscopy images of the encapsulation of pyranine in PI‐*b*‐PEG polymersomes (DIC and corresponding fluorescence channel, scale bar 50 µm) (d). Pyranine particle count after encapsulation into PI‐*b*‐PEG polymersomes using varying sonication amplitudes as well as sonication times (PI/PEG 1.99), mean + SD (*n* = 3 images per condition) (e). Cryo‐TEM images (top, scale bar: 200 nm) and cryo‐SEM images (bottom, scale bar: 1 µm) of PI‐*b*‐PEG vesicles (PI/PEG 1.99) (f).

In the last decades, polymeric vesicles (i.e., polymersomes) have emerged as synthetic analogs of liposomes, drawing considerable attention in the scientific community.^[^ ^21^ ^]^ Being made up of synthetic amphiphiles, these vesicular systems are often claimed superior to liposomes both in tunability and stability.^[^ ^22^, ^23^ ^]^ Polymersomes are investigated in a variety of applications such as drug delivery,^[^ ^24^, ^25^ ^]^ imaging,^[^ ^26^ ^]^ and as nanoreactors^[^ ^27^ ^]^ amongst others. In a recent study, polymeric vesicles comprising the amphiphilic diblock poly(styrene)‐*block*‐poly(ethylene glycol) (PS‐*b*‐PEG) were shown to effectively capture ammonia in solutions simulating the intestinal fluids, whereas liposomes were destabilized under these conditions.^[^ ^20^ ^]^ However, due to the high glass transition temperature of the PS block (*T*_g_ = 378–382 K),^[^ ^28^ ^]^ this polymersome system was characterized by rather slow uptake kinetics for larger NH_3_ analogs.^[^ ^29^ ^]^ Restricted diffusion across the glassy polymersome membrane might play a critical role in these observations.

To address this issue, we hypothesized that a polymersome system, consisting of an amphiphile with a hydrophobic block of rather low *T*_g_ might accelerate and conclusively increase the diffusion of TMA across the membrane, allowing its application for the symptomatic treatment of TMAU. In order to verify this hypothesis, we selected poly(isoprene)‐*block*‐PEG (PI‐*b*‐PEG) as amphiphile (*T*_g,PI_ = 204–209 K)^[^ ^30^ ^]^ and synthesized polymers with PI/PEG (w/w) ratios varying between 1 and 4, and a PEG fragment of 2000 g mol^−1^, via nitroxide‐mediated polymerization (NMP)^[^ ^31^ ^]^ (**Table** **1**).

**Table 1:** Characteristics of PI‐*b*‐PEG amphiphiles and polymersomes prepared by the emulsification/sonication method under optimized conditions

PI/PEG [w/w]	*M*_n_ [g mol^−1^]^a)^	*M*_n_ [g mol^−1^]^b)^	*Ð* ^b)^	*d* [nm]^c)^	PDI^c)^	*d* [µm]^d)^	Span^d)^
1.32	4600	11 100	1.25	240	0.26	n.d.	
1.99	6000	13 700	1.28	*		7.33	1.63
2.30	6600	14 400	1.30	*		5.10	1.11
2.90	7800	16 700	1.25	*		8.52	2.43
3.62	9200	19 600	1.27	*		2.79	0.86

a) Calculated by ^1^H NMR spectroscopy

b) Measured by SEC in THF

c) Obtained by DLS, polydispersity index (PDI)

d) Obtained by LD; ^*^diameter >800 nm; n.d.: not detectable.

We investigated these polymers for their ability to form giant polymersomes (in the low micrometer range, to prevent any permeation of polymersomes through the skin) at concentrations that would make them compatible with pharmaceutical applications. While the self‐assembly of amphiphiles has been extensively studied and various methods for the preparation of polymersomes have been reported,^[^ ^32^, ^33^ ^]^ there are only scarce reports describing supramolecular structures formed by PI‐*b*‐PEG in aqueous media, focusing mainly on micellar and hybrid vesicular structures.^[^ ^31^, ^34^, ^35^, ^36^, ^37^ ^]^ We screened the ability of PI‐*b*‐PEG to form polymersomes via light/fluorescence microscopy assay using three preparation methods, namely emulsification by sonication, nanoprecipitation, and film rehydration (Figure 1b). In a first step, the amphiphiles' ability to form round‐shaped structures was investigated under various conditions in order to exclude nonspherical structures, such as irregular aggregates (e.g., pointed with arrows for PI/PEG 3.62 (w/w) in Figure 1c). Subsequently, conditions leading to spherical shaped microparticles were tested for their capacity to trap and retain the hydrophilic dye pyranine. Owing to its negative charge, pyranine does not adsorb to hydrophobic matrices and can therefore be used to indirectly monitor polymersome formation.^[^ ^38^ ^]^ Encapsulation of pyranine varied between samples, but increasing the PI/PEG weight ratio (and therefore the length of the hydrophobic block) from 1.32 to 1.99 improved entrapment (Figure 1d). By increasing the PI/PEG ratio even further, encapsulation decreased to a point when no dye could be encapsulated (PI/PEG 3.62). The uptake of pyranine was lower in case of film rehydration method compared to the nanoprecipitation and emulsification/sonication procedure (Table S1 and Figure S1, Supporting Information). Furthermore, with the emulsification/sonication procedure, it was possible to obtain a higher yield of polymersomes (Table S1, Supporting Information). Hence, we selected and further optimized this method for subsequent experiments, using the polymer showing the highest encapsulation efficiency (PI/PEG 1.99) (Figure 1e; Figure S2, Supporting Information). This way, it was found that the conditions resulting in a high polymersome yield were those of low energy input, i.e., short sonication time (1 min) under a mild amplitude (5%). These conditions were then employed to prepare polymersomes with all synthesized amphiphiles. The polymersomes were characterized by dynamic light scattering (DLS) as well as laser diffraction (LD) measurements (Table 1). Most vesicles had a diameter in the lower micrometer range, which was desirable to prevent any penetration of polymersomes across the skin or mucosa. Size, morphology, and vesicular structure were analyzed by electron microscopy (EM) measurements (Figure 1f). Whilst confirming the vesicular structure, cryogenic transmission EM (cryo‐TEM) as well as cryogenic scanning EM (cryo‐SEM) experiments revealed the majority of polymersomes to exhibit smaller sizes (100–600 nm), which is possibly due to overestimation of larger structures by LD (volume distribution) and the fact that the LD analysis does not allow for a differentiation between individual and aggregated vesicles. Further, we evaluated the stability of the ester bond connecting the two polymer blocks towards hydrolysis by SEC, after one month of storage in citric acid buffer (pH = 2.0) at 4 °C (Figure S3, Supporting Information). Only a slight change at higher retention volumes could be observed, which might however be related to the presence of residual citric acid.

We then investigated the ability of the polymersome suspensions to sequester TMA in vitro using side‐by‐side diffusion cells.^[^ ^18^ ^]^ The experiments were initially performed using previously reported conditions^[^ ^29^ ^]^ at pH 6.8 to confirm that the uptake kinetics of PI‐*b*‐PEG polymersomes (inner pH 2.0) would be improved versus PS‐*b*‐PEG. Both polymers had equivalent PEG (2000 g mol^−1^) and similar hydrophobic/hydrophilic weight ratios (PI‐*b*‐PEG 2.30 and PS‐*b*‐PEG 2.15). Polymersomes comprised of PI‐*b*‐PEG did indeed exhibit faster TMA uptake kinetics (initial slope of 230 µmol g^−1^ h^−1^ vs 60 µmol g^−1^ h^−1^ in the first 2 h) and higher encapsulation efficiency (530 µmol g^−1^ vs 240 µmol g^−1^ after 24 h) as compared to those made of PS‐*b*‐PEG (**Figure** **2**a). The enhanced performance of PI‐*b*‐PEG vesicles might be related to the lower *T*_g_ of the PI block, allowing for faster diffusion of TMA across the bilayer membrane. In both cases, a slight decrease in capture capacity after 24 h could be observed, likely related to the leakage of TMA following an increase of the inner core osmolarity.^[^ ^39^ ^]^

**Figure 2:**
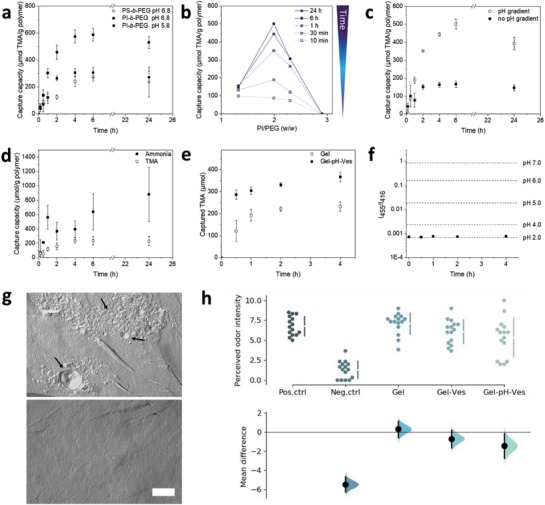
TMA capture over time for transmembrane pH gradient PS‐*b*‐PEG (PS/PEG 2.15) and PI‐*b*‐PEG (PI/PEG 2.30) polymersomes (a). Uptake capacity measured over 24 h incubation as a function of PI/PEG weight ratio (b). TMA capture over time for PI‐*b*‐PEG (PI/PEG 1.99) in the presence and absence of a pH gradient (c). TMA capture over time in the presence of tenfold excess NH_3_ (d). TMA capture of PI‐*b*‐PEG HEC hydrogel (Gel‐pH‐Ves) and HEC hydrogel (Gel) at pH 5.8 (e). Fluorescence intensity ratio *I*_455_/*I*_416_ (λ_em_ 515 nm) of pyranine‐containing pH gradient polymersomes (PI/PEG 1.99) in HEC hydrogel over time and free pyranine at indicated pH values, mimicking the incubation conditions used in (e) (f). Freeze fracture replica TEM of PI‐*b*‐PEG polymersomes (PI/PEG 1.99) in the hydrogel matrix (top) and of the vesicle‐free HEC gel (bottom), scale bar is set to 500 nm (g). Results of the in‐human olfactory study. The mean difference for 4 comparisons against the shared control “Pos.ctrl” are shown in the above Cumming estimation plot. The raw data are plotted on the upper axes with the line break denoting the mean value of each group and the lines indicating the standard deviation. On the lower axes, mean differences are plotted as bootstrap sampling distributions. Each mean difference is depicted as a dot. Each 95% confidence interval is indicated by the ends of the vertical error bars (Gel: HEC gel; Gel‐Ves: HEC gel containing polymersomes without pH gradient; Gel‐pH‐Ves: HEC gel containing polymersomes with transmembrane pH gradient) (*n* = 15) (h). For panels (a,) (c), (d), and (e) data are represented as means ± SD (*n* = 3). For panels (a), (c), and (e) statistics were performed on the AUC_0‐4h_ (Table S2, Supporting Information).

Since a major part of TMA in patients is secreted in the form of sweat, a topical application of a hydrogel containing PI‐*b*‐PEG polymersomes with a pH close to that of human skin (pH 5.8)^[^ ^40^ ^]^ was considered as a potentially suitable option for the symptomatic treatment of TMAU. We therefore conducted additional uptake experiments with polymersome suspensions at pH 5.8 (Figure 2a). Under these conditions, the overall capture of TMA was 50% lower than at pH 6.8, likely due to the reduction of the pH gradient. Nonetheless, the uptake kinetics remained fast, showing an initial slope of 120 µmol g^−1^ h^−1^ versus 230 µmol g^−1^ h^−1^ within the first 2 h, followed by a plateau.

At pH 5.8, PI‐*b*‐PEG polymersomes with a PI/PEG ratio of 1.99 exhibited the highest TMA capture (392 µmol g^−1^ after 24 h, corresponding to 31% of total TMA) as compared to all other PI/PEG ratios tested (Figure S4, Supporting Information), confirming the observations of the pyranine experiments. This is also seen in Figure 2b when plotting the TMA capture capacity at various time points as a function of the PI/PEG ratio. Considering that for a given polymer mass, only the polymer present in the form of polymersomes can contribute to TMA capturing, these results indicate that the fraction of polymer in the form of polymersomes increases with the PI block length for PI/PEG ratios up to 1.99 and decreases again for higher ratios, most likely due to more pronounced polymer aggregation in the latter case (Figure 1c). Due to its superior TMA capture capacity, all subsequent experiments were carried out using the polymer with a PI/PEG ratio of 1.99. To ensure that TMA capture was indeed driven via the transmembrane pH gradient, we performed a control experiment with polymersomes having the internal pH adjusted to 5.8 (no gradient) (Figure 2c). Surprisingly, the TMA capture of these polymersomes still reached approximately 40% of that of the pH gradient system. Such an effect was not observed with ammonia (Figure S5, Supporting Information). We suspect that this may be related to the compatibility of unprotonated TMA with the PI membrane. In fact, calculation of solubility parameters for TMA and PI provided almost identical values with a difference of only 0.6 (MJ m^−3^)^½^ (Table S3, Supporting Information), indicating miscibility of the two components.^[^ ^41^ ^]^ For ammonia the difference in solubility parameters was of 13.9 (MJ m^−3^)^½^, largely above 5 (MJ m^−3^)^½^, suggesting unfavorable interactions.

As the formulation is intended to be applied on the skin, where ammonia is also present,^[^ ^42^ ^]^ we performed a competitive uptake experiment in buffer containing both ammonia and TMA (10:1 molar ratio). An uptake kinetic in favor of ammonia (which is more abundant than TMA) would bear the risk of rapidly exhausting the gradient, thereby impairing the capture of TMA despite the higher p*K*_a_ value of the latter (p*K*_a_ 9.8 vs 9.25 for ammonia).^[^ ^43^ ^]^ As shown in Figure 2d, although ammonia was preferentially taken up, TMA capture reached 60% of the level achieved without ammonia.

Following the TMA capture experiments for polymersome suspensions in aqueous buffer, we moved to the incorporation of polymersomes in hydrogel formulations. To obtain a hydrogel, hydroxyethyl cellulose (HEC) was used as a thickening agent, given its presence in several commercial pharmaceutical dosage forms. A HEC content of 2% was selected, resulting in optimal texture and spreadability characteristics for topical administration. The formulations comprising a higher mount of HEC (4% and 8%; Figure S6, Supporting Information) proved noticeably more viscous and difficult to handle. Stability of the vesicular structures in the gel matrix was confirmed by freeze fracture replica TEM, showing structures similar to those obtained in solution (Figure 2g). Vesicles seemed to form aggregates in the gel matrix, which was also confirmed by cryo‐SEM measurements (Figure S7, Supporting Information), possibly as a result of the manual mixing process when producing the formulation. However, the incorporation of the polymersomes in the gel at a concentration of 7.21 mg mL^−1^ did not have a major impact on its rheological behavior, with only a slight increase in viscosity (Figure S8, Supporting Information).

In order to mimic the in vivo conditions following a topical administration of the gel containing polymersomes, we assessed TMA capture with Franz diffusion cells (Figure 2e). The control formulation containing no polymersomes decreased the TMA concentration in the donor compartment due to the diffusion of TMA into the hydrogel. However, the TMA uptake was significantly higher and faster with the polymersome‐containing gel, capturing the majority of TMA within the first 30 min. At 4 h, a capture capacity of 200 ± 50 µmol TMA g^−1^ polymer, upon correction for TMA diffusion, was measured (Figure S9, Supporting Information) (2.2‐fold lower than the results obtained for the polymersome suspensions in side‐by‐side diffusion cells; Figure S4, Supporting Information). This reduced uptake might be attributed to the fact that in Franz cells, the receptor compartment is not stirred and thereby the diffusion of TMA is slower. To evaluate stability of the pH gradient in the gel matrix, we prepared a gel (pH_out_ = 7.0) with polymersomes containing pyranine in their acidic lumen (pH_in_ = 2.0). The fluorescence spectrum of pyranine is known to be pH dependent, wherefore stability of the gradient can be monitored by following the fluorescence excitation spectrum over time. Comparison of the ratio of the wavelengths at maximal pH dependency (455 nm) and the isosbestic point (416 nm) to those of the free dye at set pH values can be used to determine a change in pH (Figure S10, Supporting Information). In case of the hydrogel, the ratio remained constant over 4 h at 37 °C, at a value equivalent to the inner pH of the vesicles, indicating stability of the pH gradient (Figure 2f).

Following the promising in vitro data, we conducted a double blind olfactory study using an artificial skin substrate to assess the formulations' ability to lower the characteristic TMA smell upon application of the polymersome‐loaded hydrogel. Sixteen healthy volunteers were asked to assess the odor of three different formulations in comparison to two controls. In a first step, detection and recognition thresholds were defined by asking the volunteers to smell a range of samples with increasing TMA concentrations. It is to note that roughly 7% of the population is anosmic to the smell of TMA.^[^ ^44^ ^]^ In our case, one of the volunteers was found anosmic to TMA and was therefore excluded from the study. Thresholds of 62.5 ± 33.1 µmol L^−1^ for the detection and 188 ± 137 µmol L^−1^ for the recognition of TMA were established (Table S4, Supporting Information), which is in accordance with previous findings employing a similar setup.^[^ ^45^ ^]^

In the second part of the study, each individual received a total of 15 samples to assess the TMA odor, comprising three gel formulations (no polymersomes, with polymersomes—no pH gradient, with polymersomes—with pH gradient), as well as positive and negative (no TMA) buffer controls. After 3 h of incubation in a 1 × 10^−3^ m TMA‐containing buffer, the skin substrate was treated with the gel and incubated for 30 min at 37 °C. The herein used TMA concentrations exceeded those usually encountered in healthy humans (≈5 µm in human plasma),^[^ ^46^ ^]^ to ensure recognition of the distinctive TMA smell by the participants. Perceived odor intensity was rated on a ten‐point odor detection scale, ranging from 0, which equaled the negative control, up to 10, equaling the positive control (Figure 1b). Due to the subjective nature of rating the perceived odor, an expected variability of the results was observed. Nevertheless, a clear trend was detected when comparing the bootstrap sample distributions of the three HEC formulations (Figure 2h). The control gel devoid of polymersomes did not decrease the perceived odor intensity. While the formulation containing the polymersomes without pH gradient exhibited a slight decrease in TMA odor intensity (as would be expected from the uptake study in Figure 2c), only the formulation prepared with the pH gradient polymersomes was significantly different from the positive control and the control gel (Table S5, Supporting Information).

In conclusion, a series of PI‐*b*‐PEG polymers with varying chain length were synthesized and formulated into polymersomes. For the first time, synthetic polymersomes were investigated for the palliative treatment of TMAU. The approach is based on the incorporation of transmembrane pH gradient PI‐*b*‐PEG polymersomes in a topical hydrogel formulation. Owing to their fluid membrane at body temperature, these polymersomes were able to efficiently and rapidly capture TMA in vitro and, as a result, reduced perceived odor intensity when applied on a skin surrogate. This study further highlights the potential of polymersomes in detoxification applications and provides a compelling example for the use of microvesicles to sequester target molecules in addition to their conventional use in drug delivery.

## Experimental Section

##### Polymers

PI‐*b*‐PEG and PS‐*b*‐PEG polymers were synthesized via NMP and atom transfer radical polymerization (ATRP), respectively (see the Supporting Information),^[^ ^20^, ^31^ ^]^ and characterized by ^1^H NMR spectroscopy (Figure S11, Supporting Information) and size exclusion chromatography (SEC; Figure S12, Supporting Information). ^1^H NMR spectra were recorded on an AV‐400 400 MHz spectrometer (Bruker, Billerica, MA) at room temperature, using CDCl_3_ or acetone‐d6 as solvent. Chemical shifts are reported in parts per million (ppm) and were adjusted to the corresponding solvent peak. Size exclusion chromatograms were obtained on a Viscothek TDAmax system (Viskotek, Houston, TX) equipped with a differential refractive index detector (TDA 302, Viskotek) and two ViscoGEL columns (GMH_HR_‐M, poly(styrene‐*co*‐divinylbenzene)), using tetrahydrofurane (THF) as organic phase. Samples were dissolved in THF and measured at a flow rate of 0.5 mL min^−1^. Results were obtained by comparison to a poly(methyl methacrylate) standard curve (2500–89 300 g mol^−1^) (PSS polymer, Mainz, Germany).

##### Polymersome Preparation Film Rehydration

Polymers were dissolved in dichloromethane (DCM) (3 mg in 200 µL), added into a 4 mL glass vial and subsequently dried to obtain a thin polymer film. The film was then rehydrated using sodium chloride‐containing citrate buffer (1 mL, 250 × 10^−3^ m citric acid, pH = 2, 300 mOsmol kg^−1^) and stirred for 3 days at room temperature.

##### Nanoprecipitation

Polymers were dissolved in THF (4 mg in 333 µL) and added dropwise to sodium chloride‐containing citrate buffer (1 mL, 250 × 10^−3^ m citric acid, pH = 2, 300 mOsmol kg^−1^), whilst stirring at room temperature for 5 min. Excess solvent was removed in vacuum (1 h, 40 °C, 15 kPa).

##### Emulsification

Polymers (PI‐*b*‐PEG or PSn‐*b*‐PEG) were dissolved in DCM (30 mg in 100 µL) and added to sodium chloride‐containing citrate buffer (1 mL, 250 × 10^−3^ m citric acid, pH = 2, 300 mOsmol kg^−1^) dropwise, whilst sonicating using a FB‐705 sonic dismembrator (Thermo Fisher Scientific, Waltham, MA) (Optimized conditions: sonication for 1 min at an amplitude of 5). The sample was cooled with an ice bath during this process. Excess solvent was removed in vacuum (7 min, 40 °C, 65 kPa).

##### Encapsulation of Pyranine

In the case of pyranine containing samples, vesicles were prepared as mentioned above, exchanging the citrate buffer to miliQ water containing 10 × 10^−3^ m of pyranine. Free pyranine was removed using PD Miditrap G‐25 size exclusion columns (GE healthcare, Uppsala, Sweden), applying the following protocol: after conditioning the column with water, the sample (200 µL) was applied. Water (800 µL), followed by another fraction of water (600 µL) were added, of which the latter was collected. Polymersome samples were stored for up to 4 days post preparation at 4 °C.

##### Polymersome Characterization

Polymer concentration: Polymer concentrations in the polymersome suspensions were determined by lyophilization of polymersome‐containing samples (75 µL), subsequent dissolution in THF (1 mL), and measurement by SEC. Peak integrals were compared to those of a subset of standards of the polymer used to obtain the polymersomes (0.1, 0.2, 0.5, 1, and 2 mg mL^−1^).

##### Size Measurements

Hydrodynamic diameters were determined by dynamic light scattering (DLS) on a DelsaNano (Beckman Coulter, Indianapolis, IN) using the cumulant method. For samples exceeding a diameter of 800 nm, additional measurements using laser diffraction (LD) were performed on a Malvern MasterSizer2000 (Malvern Instruments, Malvern, UK), reporting results as volume distribution.

##### Optical Microscopy

Microscopy images were obtained on a Leica DMI6000B inverted epifluorescence microscope (Leica Microsystems, Wetzlar, Germany) at room temperature. Images were analyzed using ImageJ and particle counting was performed in MatlabR2018b using the Image Processing and Computer Vision toolbox. Code is available upon request.

##### Electron Microscopy

Samples were analyzed by cryo‐TEM, cryo‐SEM, and freeze fracture replica TEM (see the Supporting Information).

##### In Vitro Uptake Assays‐TMA/Ammonia Uptake in Phosphate Buffer

Uptake experiments of PI‐*b*‐PEG (or PS‐*b*‐PEG) polymersomes were conducted in side‐by‐side diffusion cells (PermeGear, Hellertown, PA) as described elsewhere.^[^ ^18^ ^]^ Briefly, two 3.4 mL chambers, separated by a 0.1 µm polycarbonate membrane filter, were prepared by mixing sodium chloride‐containing phosphate buffer at chosen pH (5.8 or 6.8) (2.8 mL, 60 × 10^−3^ m in phosphate, 300 mOsmol kg^−1^) and 2 M NaOH (125 µL for pH 5.8, 145 µL for pH 6.8) in each chamber, to neutralize the citrate buffer that was added in the subsequent step and ensure a final pH_out_ of 5.8 or 6.8). Polymersome suspension in citrate buffer (400 µL, 10.2 mg mL^−1^) and sodium chloride‐containing citrate buffer (400 µL, 250 × 10^−3^ m citric acid, pH = 2, 300 mOsmol kg^−1^) were added to the receptor and donor cells, respectively. Water was added to obtain the final volume of 3.4 mL in each cell. After incubation at 37 °C for 15 min, a 100 × 10^−3^ m TMA (or NH_4_Cl) solution (25.5 µL) was added to both chambers to start the capture experiment. Samples (50 µL) were drawn at regular time intervals from the donor cell. The TMA content was determined as described previously with minor modifications.^[^ ^46^ ^]^ In short, calibrator (50 µL) or TMA samples were mixed with internal standard (50 µL) and liberation solution (900 µL, 2 m NaOH/ 0.5 m KCl) in a 20 mL headspace vial and capped directly. Calibration was performed using single point calibration at 500 × 10^−6^ m. Ammonia concentration of samples was determined with the Berthelot assay.^[^ ^20^ ^]^

##### Ammonia and TMA Competition Experiment

The competition experiment was conducted in the same fashion as the TMA capture experiment, adding a ten‐time excess of NH_4_Cl (7.5 × 10^−3^ m) with respect to TMA.

##### Hydrogel Formulation and TMA Capture

HEC (4.0 g) was weighed into a 50 mL cream container and sodium chloride‐containing phosphate buffer mixture was added (46 g, 60 × 10^−3^ m, pH = 5.8, 300 mOsmol kg^−1^). The mixture was blended using an Unguator Q device (Gako International, Schesslitz, Germany). Immediately after mixing, polymersome suspension (9 g, 16 mg mL^−1^) in citrate buffer were added to 10 g of the freshly prepared gel. NaOH 10 m was added to reach a final pH of 5.8, and then milliQ water was added to reach a total weight of 20 g. The ingredients were manually mixed and stored at 4 °C for 2 days to obtain the final hydrogel formulation. Polymersome‐free gel was prepared the same way, employing citrate buffer without the polymersomes. TMA capture of PI‐*b*‐PEG hydrogels was investigated in Franz cells (PermeGear), separating buffer and sample chamber using a 0.1 µm polycarbonate membrane filter. Approximately 1 g of gel formulation was added to the sample chamber. After addition of sodium chloride‐containing phosphate buffer (60 × 10^−3^ m in phosphate, pH = 5.8, 300 mOsmol kg^−1^), 0.75 × 10^−3^ m in TMA, capture was evaluated at 37 °C, drawing samples (50 µL) at regular time intervals.

##### Stability Assessment of pH Gradient in HEC gel

The stability of the pH gradient of the polymersomes in the hydrogel matrix was assessed by encapsulation of pyranine into the vesicles as described previously, using sodium chloride‐containing citrate buffer (250 × 10^−3^ m citric acid, pH = 2, 300 mOsmol kg^−1^) instead of milliQ water, and subsequent preparation of the vesicle containing hydrogel (pH_inside_ = 2.0, pH_outside_ = 7.0). Fluorescence excitation spectra (λ_em_ 515 nm) were recorded and subsequent comparison of the intensity ratio between λ_exc_ 455 nm (maximum pH dependency) and λ_exc_ 416 nm (isosbestic point) to those of a series of pyranine solutions (20 × 10^−6^ m) at set pH values was performed over time.

##### In‐Human Study

The protocol for the in human study was approved by the Research Ethics Committee of ETH Zurich (EK 2018‐N‐74). Informed consent of all participating subjects was obtained. A more detailed version of the protocol can be found in the supplementary information.

##### Threshold Testing

Volunteers were asked to smell and evaluate a range of increasing concentrations of TMA in sodium chloride‐containing phosphate buffer (60 × 10^−3^ m in phosphate, pH = 5.8, 300 mOsmol kg^−1^). Solutions were prepared in sealable glass containers of 50 mL and incubated at 37 °C for 30 min. Starting from a concentration of 15.6 × 10^−6^ m in TMA (≈1 mg L^−1^), concentration was doubled up to the point that the subject was able to detect (detection threshold) and subsequently recognize (recognition threshold) the distinct smell of TMA.

##### In‐Human Evaluation of PI‐*b*‐PEG Formulation

The olfactory evaluation of the hydrogel formulation was performed in a double blind fashion. Artificial skin substrate (VitroSkin, IMS, Bunnell, FL) was incubated in sodium chloride‐containing phosphate buffer (60 × 10^−3^ m, pH = 5.8, 300 mOsmol kg^−1^), 1 × 10^−3^ m in TMA, for 3 h (in case of negative controls phosphate buffer without TMA was used). The substrate was subsequently placed on a glass slide, treated with the HEC formulation (≈200 mg), enclosed in a 50 mL falcon tube, and incubated at 37 °C for 30 min. Positive and negative controls, were incubated in the absence of HEC formulation. Samples were rated on a scale of 1 to 10, increasing in TMA smell. Each individual received a total of 15 samples (3 times each: positive control, negative control, PI‐*b*‐PEG HEC gel with pH gradient – Gel‐pH‐Ves, PI‐*b*‐PEG HEC gel without pH gradient – Gel‐Ves, and pure HEC gel – Gel) in a randomized fashion. Time between samples was 10 min to ensure recovery of the olfactory receptors.

##### Statistical Analysis

Statistical analysis was carried out using GraphPad Prism (version 8.2.0). In the case of the olfactory study, the groups were compared using a one‐way ANOVA (Tukey's test, paired), assuming normal distribution of the data. Estimation graphics were plotted using the DABEST Python package in Python 3.6.^[^ ^47^ ^]^ In case of TMA and ammonia in vitro capture assays, statistical analysis was performed on the area under the uptake versus time curve for the first 4 h (AUC_0‐4h_). Multiple groups were compared using a one‐way ANOVA (Tukey's test, unpaired), whereas groups of two were compared using an unpaired t test.

## Conflict of Interest

The authors declare no conflict of interest.

## Supporting information

Supporting Information
