# Supplementary material for: Engineered Polymersomes for the Treatment of Fish Odor Syndrome: A First Randomized Double Blind Olfactory Study
Source: Adv Sci (Weinh). 2020 Mar 9;7(8):1903697. doi: 10.1002/advs.201903697 (PMC7175261; doi:10.1002/advs.201903697)
Supplement: Supplementary file 1 — Supporting Information [file ADVS-7-1903697-s001.pdf]

Copyright WILEY-VCH Verlag GmbH & Co. KGaA, 69469 Weinheim, Germany, 2020.

ADVANCED SCIENCE
Open Access

Supporting Information

for *Adv. Sci.,* DOI: 10.1002/advs.201903697

Engineered Polymersomes for the Treatment of Fish Odor Syndrome: A First Randomized Double Blind Olfactory Study

*Aaron C. Schmidt, Erik R. Hebels, Charlotte Weitzel, Anna Kletzmayr, Yinyin Bao, Christian Steuer, and Jean-Christophe Leroux**

Copyright WILEY-VCH Verlag GmbH & Co. KGaA, 69469 Weinheim, Germany, 2020.

Supporting Information

Engineered polymersomes for the treatment of fish odor syndrome: A first randomized double blind olfactory study

*Aaron C. Schmidt, Erik R. Hebels, Charlotte Weitzel, Anna Kletzmayr, Yinyin Bao, Christian Steuer, and Jean-Christophe Leroux**

Materials

Isoprene, styrene, sodium hydroxide, ammonium chloride, 2-bromopropionyl bromide, potassium dihydrogen phosphate, sodium sulfate, sodium bicarbonate, tridosium citrate dihydrate, phenol nitroprusside, alkaline hypochlorite, aluminum oxide basic and hydroxypyrene-1,3,6-trisulfonic acid trisodium salt (pyranine) were purchased from Sigma Aldrich (Steinheim, Germany). Poly(ethylene glycol) (PEG) monomethyl ether (M_n = 2000 Da) was purchased from Fluka (Buchs, Switzerland). Copper powder and *N*,*N*,*N*,*N*,*N*-pentamethyldiethylenetriamine (PMDETA) were obtained from Acros (Geel, Belgium). Triethylamine was obtained from Merck (Darmstadt, Germany). Copper^{I} bromide was supplied by Alfa Aesar (Kandel, Germany). Sodium chloride and citric acid monohydrate were purchased from Fisher Scientific (Leicester, U.K.). SilicaFlash P60 (40-63 µm, 230-400 mesh) was purchased from SiliCycle (Quebec City, QC, Canada). Trimethylamine (TMA) and 4,4'-dinonyl-2,2'-dipyridyl were obtained from TCI (Zwijndrecht, Belgium). Deuterated chloroform ($CDCl_3$) was purchased from Armar (Dottingen, Switzerland). Hydroxyethyl cellulose 5000 was obtained from Hänseler AG (Herisau, Switzerland). Solvents were obtained from VWR, Fisher Scientific, Acros, and Sigma Aldrich. 2,2,5-trimethyl-4-phenyl-3-azahexane-3-nitroxide (TIPNO)[1], PEG_{2000}-Br and PEG_{2000}-*b*-PS were synthesized according to literature.[2]

Synthesis of poly(ethylene glycol) monomethyl ether 2-bromoproionate (PEG_{2000}-Br)

PEG monomethyl ether (30.3 g, 15.2 mmol, 1 eq.) was dried in vacuum for 4 h at 60 °C to remove residual traces of water. It was then dissolved in dry tetrahydrofuran (THF) (200 mL), followed by triethylamine (10.5 mL, 75.8 mmol, 5 eq.) and kept under inert atmosphere. Subsequently, 2-bromopropionyl bromide (7.95 mL, 75.8 mmol, 5 eq.) dissolved in THF (20 mL) was added dropwise whilst cooling with an ice bath. After 24 h at room temperature, the reaction was stopped, the hydrobromine salts were removed by centrifugation, and the supernatant was concentrated in vacuum. The residue was dissolved in water and extracted with dichloromethane (DCM), washed with saturated sodium bicarbonate solution and brine, and dried over sodium sulfate. After concentrating in vacuum, it was dissolved in DCM, precipitated in cold diethyl ether and dried in vacuum. The final product was obtained as a white solid (30.3 g, 93%).
^{1}H NMR (400 MHz, $CDCl_3$) δ 4.34 (q, 1H), 4.25 (t, 2H), 3.78 – 3.37 (m, 4H per PEG unit), 3.31 (s, 3H), 1.76 (d, 3H).

Synthesis of poly(ethylene glycol) monomethyl ether alkoxyamine (PEG_{2000}-TIPNO)

PEG_{2000}-Br (2 g, 0.9 mmol, 1 eq.), copper^{I} bromide (67 mg, 0.5 mmol, 0.6 eq.), copper (150 mg, 2.3 mmol, 2.6 eq.) were weighed in a Schlenk flask, sealed with a rubber septum, and

deoxygenated by three vacuum-argon cycles. Dry toluene (20 mL), PMDETA (0.4 mL, 1.9 mmol, 2.1 eq.), and TIPNO (0.8 mL, 2.2 mmol, 2.5 eq.) were added under argon and the mixture was further degassed by 3 freeze-thaw cycles and backfilled with argon. The flask was placed in a pre-heated oil bath at 80 °C and left for 48 h to react. The crude mixture was subsequently precipitated in diethyl ether and residual copper was removed using flash chromatography (20:1 chloroform/methanol). The eluent was then dried in vacuum, dissolved in DCM and precipitated in diethyl ether. Drying in vacuum yielded PEG_{2000}-TIPNO as a white solid (1.6 g, 76%).
^{1}H NMR (400 MHz, $CDCl_3$) δ 7.58 – 7.03 (m, 5H), 4.50 and 4.43 (q, 1H), 4.32 – 4.17 (m, 2H), 3.78 – 3.37 (m, 4H per PEG unit), 3.31 (s, 3H), 3.24 (d, 1H), 2.09 and 1.95 (m, 1H, diastereomers), 1.50 and 1.36 (d, 3H, diastereomers), 1.14 and 0.99 (d, 3H, diastereomers), 0.91 and 0.85 (s, 9H, diastereomers), 0.44 and 0.37 (d, 3H, diastereomers).

Polymerization of isoprene
PEG_{2000}-TIPNO macroinitiator (200 mg), xylenes (2 mL), and isoprene (eq. varying) were given into a 30 mL pressure tube and closed. The mixture was degassed by 3 freeze thaw cycles, backfilled with argon, and sealed. It was subsequently placed in a pre-heated oil bath at 125 °C and reacted for 48 h. The reaction was quenched by cooling to room temperature. Residual solvent and monomers were removed by co-evaporations with diethyl ether at 50 °C and drying in vacuum, affording a yellow semi-solid.
^{1}H NMR (400 MHz, $CDCl_3$) δ 7.36-7.21 (m, 5H C6*H*5), 5.79 – 5.59 (m, 1H per unit of 1,2-PI), 5.18 – 4.93 (m, 1H, 1,4-PI), 4.92 – 4.72 (m, 2H, 1,2-PI), 4.72 – 4.50 (m, 2H 3,4-PI), 4.17 – 4.05 (m, 2H), 3.79 – 3.35 (m, 4H 19 per unit of PEG), 3.31 (s, 3H), 2.09 – 1.84 (m, 2H, 1,4- and 3,4-PI), 1.68 – 1.45 (m, 3H, 1,4- and 3,4-PI).

Polymerization of styrene
PEG_{2000}-Br macroinitiator (2.0 mmol) was loaded in a flame-dried Schlenk flask, along with copper bromide (CuBr, 3.0 mmol) and 4,4'-dinoyl-2,2'-dipyridyl (2.64 mmol) as the catalyst and ligand, respectively. The Schlenk flask was evacuated and refilled with argon for several cycles to remove oxygen. In a separate flask, styrene (100 mmol, 11.5 mL) was deoxygenated by argon bubbling for 0.5 h, and then loaded in the Schlenk flask. The mixture was then heated at 115 °C during 16 h and the solution containing the black product was dissolved in THF, filtered through a basic alumina column and precipitated twice in hexane. The precipitate was collected and dried under vacuum.

^{1}H NMR (400 MHz, acetone-d6) δ 7.15-6.48 (m, 5H per St unit, -C6*H*5), 3.42-3.78 (m, 182H, -C*H*2C*H*2O), 3.31 (s, 3H, -OC*H*3), 1.96-1.62 (m, 3H per St unit, -C*H*2C*H*- and 6H, -C(C*H*3)2). $M_{n,\mathrm{NMR}}$ = 4100 (PI) – 2000 (PEG), $M_{n,\mathrm{GPC}}$ = 6300, $Đ$ = 1.43.

Electron microscopy measurements
Samples were analyzed in the same conditions used in the capture experiments at pH 5.8.
Cryo TEM: Five µL of each sample was applied to a gold grid covered by holey gold film (UltrAuFoil 2/1 Quantifoil Micro Tools GmbH, Jena, Germany) and excess of liquid was blotted automatically between two strips of filter paper. Subsequently, the samples were rapidly plunged into liquid ethane (cooled to ~ 180 °C) in a cryobox (Carl Zeiss NTS GmbH, Oberkochen, Germany). Excess ethane was removed with a piece of filter paper. The samples were transferred immediately with a Gatan 626 cryo-transfer holder (Gatan, Pleasanton, CA) into the pre-cooled cryo-electron microscope (Philips CM 120, Eindhoven, Netherlands) operated at 120 kV and viewed under low dose conditions. The Images were recorded with a 2k CMOS Camera (F216, TVIPS, Gauting, Germany). In order to minimize the noise, four images were recorded and averaged to one.

Cryo SEM: The samples were enclosed between 0.1-mm thick copper profiles, as performed in the sandwich double-replica technique and cryo-fixed by rapid plunge-freezing in a liquid (1 : 1) ethane–propane mixture cooled by liquid nitrogen. The samples were quickly transferred to the freeze-fracture unit ACE900 (Leica, Wien, Austria) at -150 ℃ and fractured. The revealed fracture plane was sublimed at -100 °C for 5 min. After that samples were shadowed under 45° with 2 nm platinum/carbon, stabilized by deposition of 3 nm pure carbon under 90° and transferred with the VCT100 Shuttel (BAL-TEC, Balzers, Liechtenstein) in a pre-cooled (-140 °C) field emission scanning EM (LEO1530, Zeiss, Germany). Images were recorded with in-lens detector at 4−6 kV.

Freeze fracture replica TEM: Aliquots of the sample were enclosed between 0.1-mm thick copper profiles, as performed in the sandwich double-replica technique. The sandwiches were physically fixed by rapid plunge-freezing in a liquid ethane-propane (1:1 v/v) mixture cooled by liquid nitrogen. Freeze-fracturing was performed in a BAF 400 T freeze-fracture unit (BAL-TEC, Balzers, Liechtenstein) at -150 ℃ using a double replica stage. The fractured samples were shadowed under 35° with 2 nm platinum/carbon and stabilized by deposition of 20 nm pure carbon under 90° for replica production. Replicas were cleaned with chloroform/methanol (50:50 v/v) and viewed with a transmission electron microscope (TEM, Point-Electronik, EM 900, Germany) operated at 80 kV. The Images were recorded with the 2k-CCD -Camera (TRS Tröndle, Germany).

Rheological measurements

Viscosity measurements were performed using a HAAKE RheoStress 600 rotational rheometer (Thermo Electron Corporation, Waltham, MA) with cone and plate geometry (35 mm/2°). Viscosity was determined at a temperature of 37 °C, applying an increasing shear rate of 1 to 100 s^{-1} in the first minute, keeping it at 100 s^{-1} the following minute, and decrease back to 1 s^{-1} within a minute. Data was analyzed by RheoWin Data Manager (Thermo Electron Corporation, Waltham, MA).

In human studies

A total of 16 volunteers were recruited (4 males, 12 females, ages 23–43) from the Institute of Pharmaceutical Sciences at ETH Zurich. Volunteers were fulfilling the following requirements: they were neither smokers nor allergic nor having frequent colds. They were not consuming any food or beverages other than water 2 h prior to testing. They were not using perfume, deodorant, cologne, or other fragrance cosmetics 12 h prior to the test. The test was performed in a well ventilated room.

Threshold testing: The objective was the determination of the detectable concentration range of TMA in the conditions used in the subsequent study. A range of solutions of TMA in sodium chloride-containing phosphate buffer (60 mM, 300 mOsmol kg^{-1}) at pH 5.8 was prepared, starting at a concentration of 15 µM, equaling ~ 1 mg/L, increasing twofold every step. The samples (5 mL) were prepared in 50 mL sealable glass containers, incubated at 37 °C for 30 min, and only opened prior to testing. Volunteers were asked to smell and evaluate these solutions, starting at the lowest concentration. After evaluation of the sample, a break of 2 min was taken before evaluating the next sample. This procedure was repeated at the point that volunteers were able to detect a smell (detection threshold). After a 5 min break, the next sample was evaluated. This procedure was repeated until volunteers were able to clearly recognize (recognition threshold) the distinctive smell of TMA.

Evaluation of PI-*b*-PEG formulation: This part of the study was conducted in a randomized double blind fashion. Each volunteer was asked to smell two buffer controls (negative and positive) to calibrate the olfactory system for the subsequent evaluation of 15 samples, which were provided in a randomized fashion. In between samples, a break of 10 min was taken to ensure recovery of the olfactory receptors. The 15 samples consisted of 5 sets of triplicates,

including a negative buffer control, a positive buffer control, a pure HEC gel (Gel), a vesicle containing gel without pH gradient (Gel-Ves), and a gel containing vesicles with a pH gradient (Gel-pH-Ves). In all cases, except the negative control, artificial skin substrates (VitroSkin, IMS, Bunnell, FL) were incubated in a sodium chloride-containing phosphate buffer (60 mM, 300 mOsmol kg^{-1}, pH 5.8), containing 1 mM in TMA for 3 h. For the negative control, a phosphate buffer not containing TMA was used. After incubation skin substrates were placed on glass slides, which were placed in 50 mL flacon tubes, sealed, and subsequently incubated for 30 min at 37 °C. In case of the three HEC formulations, 200 mg of gel were applied on the skin substrate after incubation in TMA containing phosphate buffer. Volunteers were asked to rate samples on a scale of 1 to 10, where 1 equaled the negative and 10 the positive buffer control.

Supplementary Figures and Tables

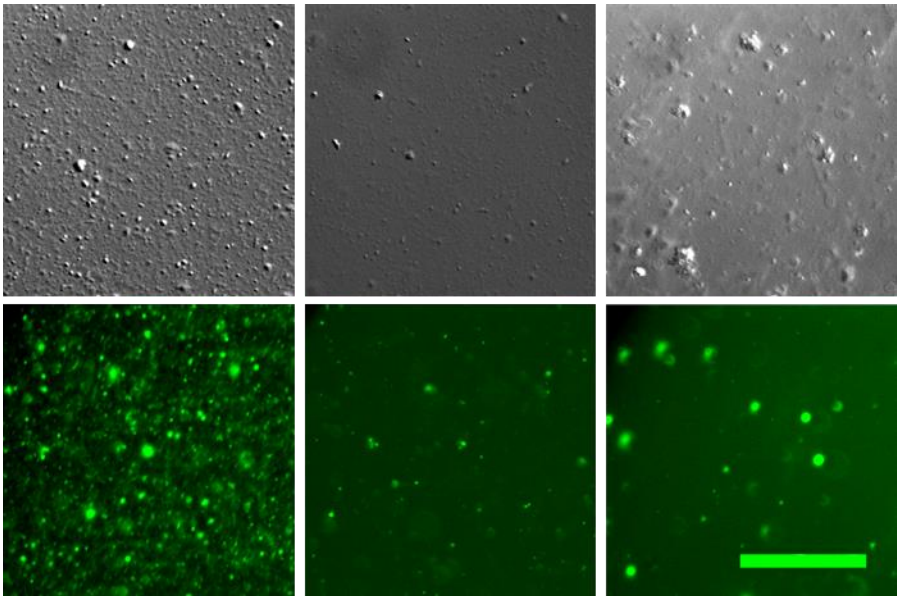

Figure S1. Fluorescence microscopy images of pyranine-containing vesicles prepared by emulsification (left), nanoprecipitation (middle), and film rehydration (right). Upper panel showing differential interference contrast (DIC) channel, lower panel showing fluorescent channel. Scale bar is set to 50 µm.

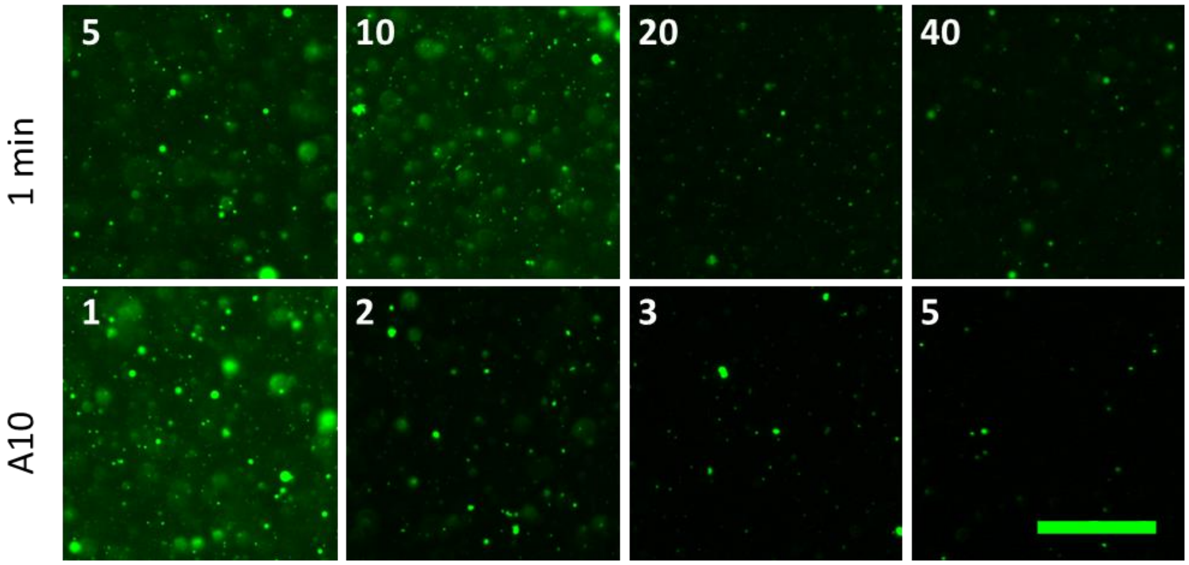

Figure S2. Fluorescence images of pyranine-containing vesicles prepared by emulsification using the lead polymer (PI/PEG = 1.99). Upper panel: Increasing sonication amplitude from left to right (indicated in left corner in %). Lower panel: increasing sonication time from left to right (indicated in left corner in min) at amplitude 10%. Scale bar is set to 50 µm.

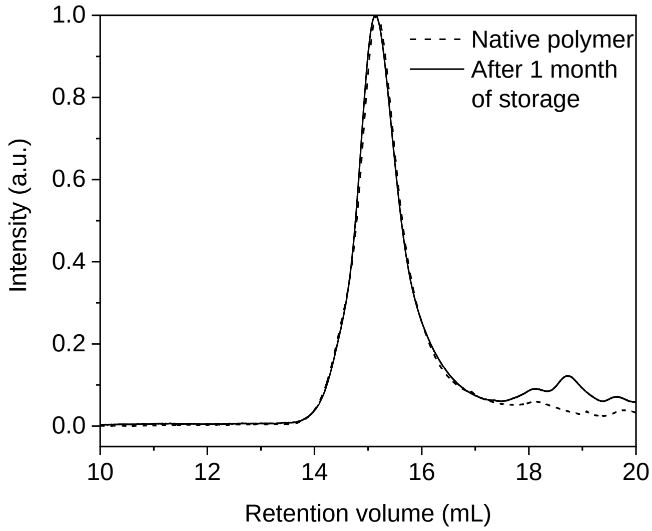

Figure S3. SEC of PI-*b*-PEG (PI/PEG 3.62) before and after 1 month of storage in citric acid buffer (pH = 2.0) at 4 °C.

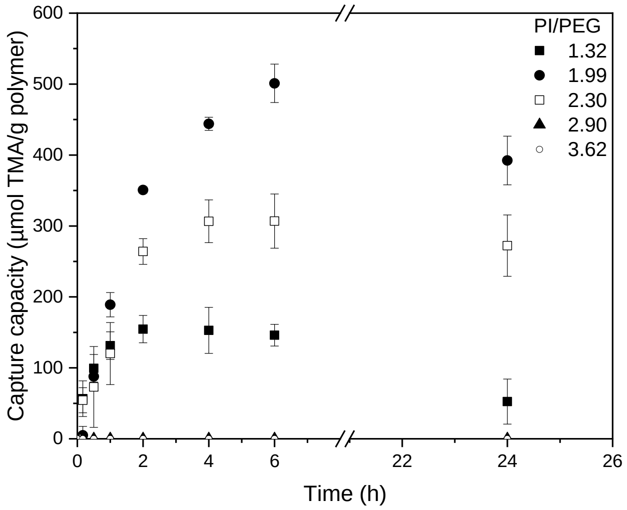

Figure S4. TMA capture over time for vesicles prepared from the various PI-*b*-PEG amphiphiles at pH 5.8, using the emulsification process (mean ± SD, n = 3). Statistics were performed on the $AUC_{0\text{-}4h}$ (see **Table S2**).

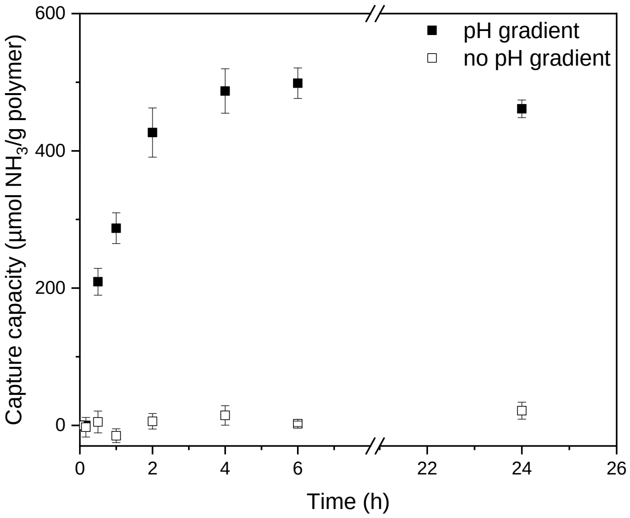

Figure S5. NH_3 capture over time for PI-*b*-PEG (PI/PEG 1.99) in the presence and absence of a pH gradient (mean ± SD, n = 3). Statistics were performed on the $AUC_{0\text{-}4h}$ (see **Table S2**).

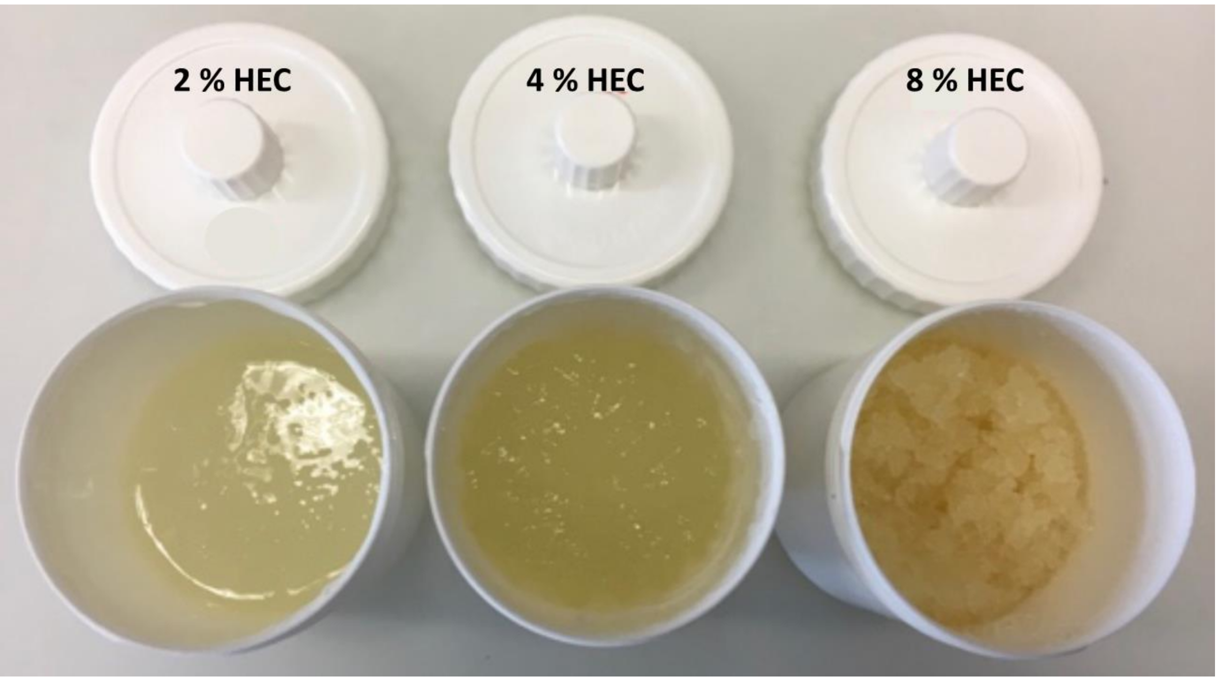

Figure S6. Hydrogels produced with an Unguator device, using the indicated HEC content in phosphate buffer.

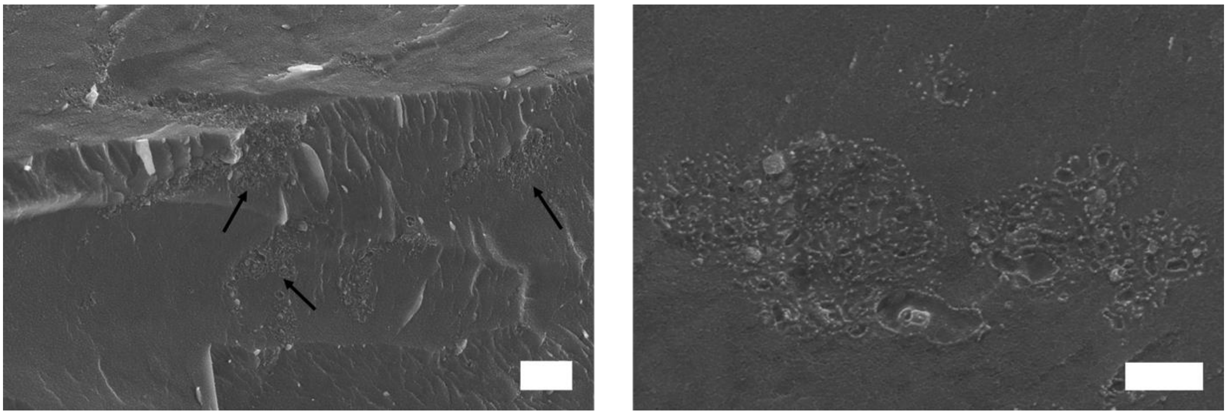

Figure S7. Cryo-SEM images of PI-*b*-PEG polymersomes (PI/PEG 1.99) in the hydrogel matrix. Scale bars set to 1 µm (left) and 0.5 µm (right).

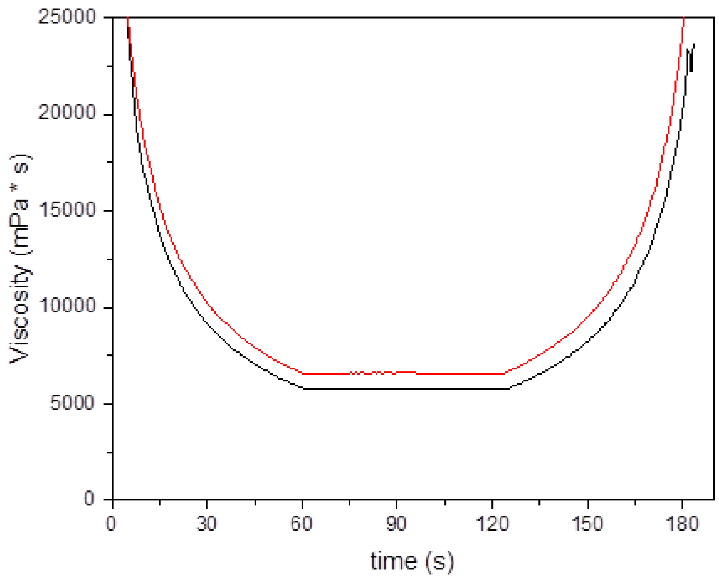

Figure S8. Rheological profiles of control HEC gel (black line) and HEC gel containing the polymersomes (PI/PEG 1.99) (red line).

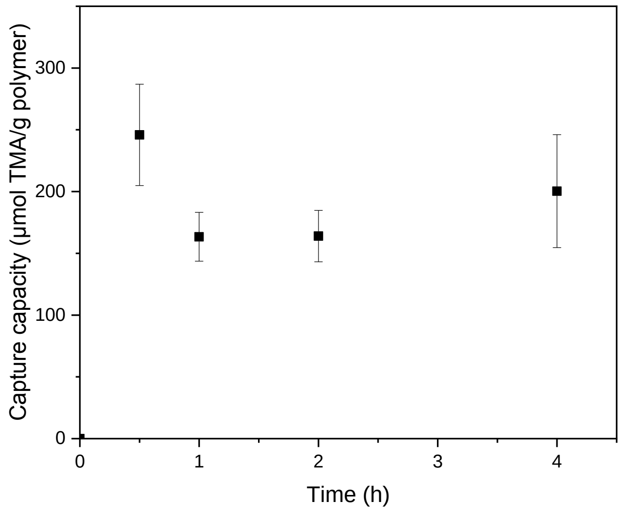

Figure S9. Capture capacity of polymersome-containing hydrogel (PI/PEG 1.99) in Franz diffusion cells at pH 5.8. Mean ± SD (n=3).

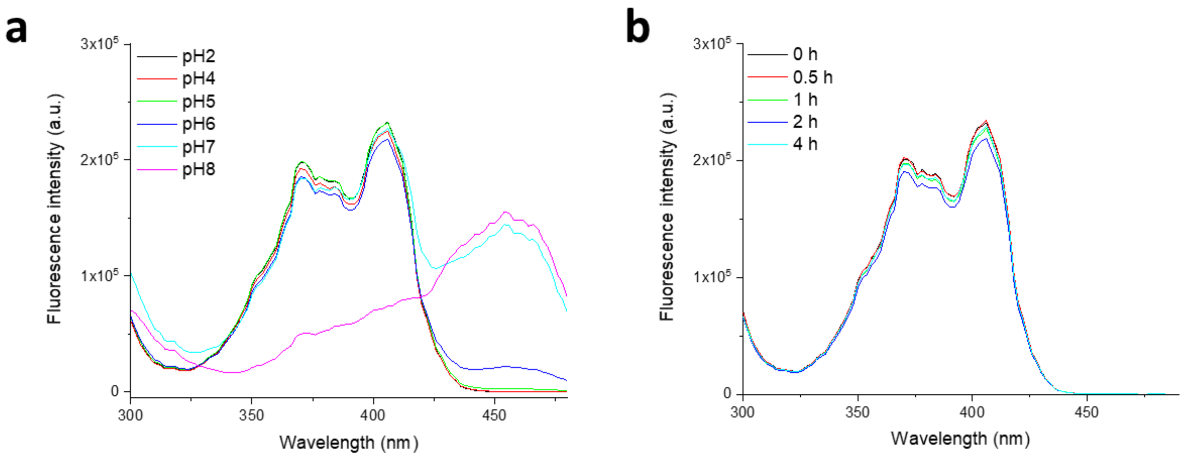

Figure S10. Fluorescence excitation spectra (λ_{em} 515 nm) of free pyranine at indicated pH values (**a**) and pyranine-containing pH gradient polymersomes (PI/PEG 1.99) at indicated times after preparation of the formulation (**b**).

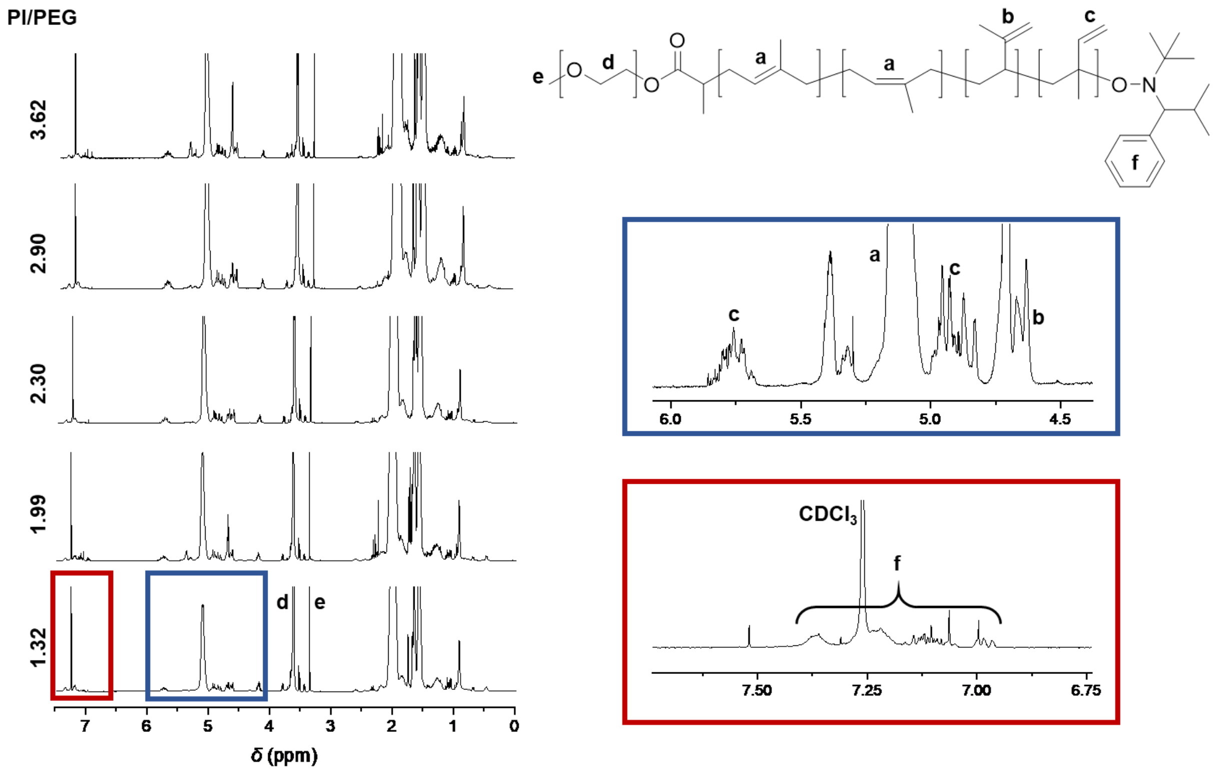

Figure S11. ^{1}H NMR of PI-*b*-PEG polymers in $CDCl_3$.

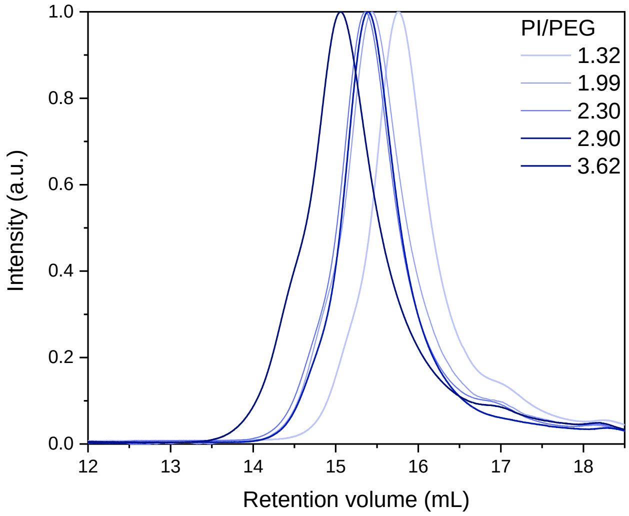

Figure S12. SEC traces of PI-*b*-PEG polymers recorded in THF at 35 °C.

Table S1. Comparison of the three vesicle preparation methods with respect to obtained polymer concentration in the suspension (n=3), and number of pyranine-containing vesicles determined by image analysis (n=4) (mean ± SD).

	PI-*b*-PEG targeted [mg mL^{-1}]	PI-*b*-PEG measured [mg mL^{-1}]	Vesicle yield [a] [%]	Pyranine count	Pyranine count normalized [b]
Emulsification	30	12.2 ± 0.7	41 ± 2	3390 ± 440	280 ± 40
Nanoprecipitation	4	3.42 ± 0.2	85 ± 4	1100 ± 60	330 ± 20
Film rehydration	3.33	3.40 ± 0.1	100	720 ± 280	210 ± 80

[a)] Vesicle yield = PI-*b*-PEG measured / PI-*b*-PEG targeted; [b)] Pyranine particle count normalized to concentration of polymer.

Table S2. Statistical data of TMA/ammonia *in vitro* capture experiments. The area under the uptake *vs.* time curves for the first 4 h ($AUC_{0\text{-}4h}$) were compared using a one-way ANOVA and subsequent Tukey's multiple comparisons test in case of multiple groups, and an unpaired t test in case of only two groups were compared.

Experiment	Mean difference [µmol g^{-1} h]	Significance	p value
PS-*b*-PEG pH 6.8 *vs.* PI-*b*-PEG pH 6.8 [b]	-990	***	0.0003
PS-*b*-PEG pH 6.8 *vs.* PI-*b*-PEG pH 5.8 [b]	-270	n.s.	0.1128
PI-*b*-PEG pH 6.8 *vs.* PI-*b*-PEG pH 5.8 [b]	720	**	0.0015
PI-*b*-PEG 1.32 *vs.* PI-*b*-PEG 1.99 [a]	-610	****	<0.0001
PI-*b*-PEG 1.32 *vs.* PI-*b*-PEG 2.30 [a]	-300	***	0.0008
PI-*b*-PEG 1.32 *vs.* PI-*b*-PEG 2.90 [a]	540	****	<0.0001
PI-*b*-PEG 1.99 *vs.* PI-*b*-PEG 2.30 [a]	310	***	0.0006
PI-*b*-PEG 1.99 *vs.* PI-*b*-PEG 2.90 [a]	1200	****	<0.0001

PI-*b*-PEG 2.30 *vs.* PI-*b*-PEG 2.90 [a]	840	****	<0.0001
PI-*b*-PEG pH gradient *vs.* PI-*b*-PEG no gradient [c]	690	***	0.0009
PI-*b*-PEG pH gradient *vs.* PI-*b*-PEG no gradient [c,d]	1690	****	<0.0001
Gel-pH-Ves *vs.* Gel [e]	470	***	0.00013

[a)] Experiment performed at pH 5.8 in side by side diffusion cells, [b)] Using PI-*b*-PEG ratio of 2.30 and PS-*b*-PEG ratio of 2.15 in side-by-side diffusion cells, [c)] PI-*b*-PEG ratio of 1.99 in side-by-side diffusion cells at pH 5.8, [d)] Capture of NH_3, [e)] PI-*b*-PEG ratio of 1.99 performed in Franz diffusion cells at pH 5.8. n.s.: not significant.

Table S3. Solubility parameters of TMA, ammonia and PI. Values were calculated using the Hoy group contribution (group values were obtained from literature).[3]

	δ $(MJ/m^3)^{1/2}$
TMA	17.4
NH_3	31.9
PI	18.0
Δ(PI, TMA)	0.6
Δ(PI, NH_3)	13.9

Table S4. Detection and recognition thresholds for TMA in phosphate buffer at pH 5.8

Subject	Age	Gender	Detection [mmol L^{-1}]	Recognition [mmol L^{-1}]
1	26	F	0.125	0.25
2	23	F	0.063	0.25
3	33	M	0.031	0.063
4	26	F	0.031	0.125
5	25	F	0.063	0.25
6	24	F	0.031	0.063
7	43	F	0.031	0.125
8	28	F	0.063	0.125
9	28	F	0.063	0.125
10	33	M	0.125	0.5
11	31	F	0.063	0.125
12	29	F	0.063	a
13	25	M	0.031	0.125
14	25	F	0.125	0.5
15	25	F	0.063	0.125
16	29	M	0.031	0.063

[a)] Subject not able to recognize the smell of TMA in any of the samples

Table S5. Statistical data of in human olfactometric study, obtained using a one-way ANOVA and subsequent Tukey's multiple comparisons test.

Subject	Mean difference [perceived odor]	Significance	P value
- *vs.* +	-5.5	****	<0.0001
- *vs.* Gel	-5.8	****	<0.0001
- *vs.* Gel-Ves	-4.8	****	<0.0001
- *vs.* Gel-pH-Ves	-4.0	****	<0.0001
+ *vs.* Gel	-0.3	n.s.	0.960
+ *vs.* Gel-Ves	0.7	n.s.	0.594
+ *vs.* Gel-pH-Ves	1.4	*	0.029
Gel *vs.* Gel-Ves	1.0	n.s.	0.218
Gel *vs.* Gel-pH-Ves	1.8	**	0.004
Gel-Ves *vs.* Gel-pH-Ves	0.7	n.s.	0.519

$^{-}$ negative control, $^{+}$ positive control, ^{Gel} HEC control gel, $^{Gel\text{-}Ves}$ HEC gel containing PI-*b*-PEG polymersomes without pH gradient, $^{Gel\text{-}pH\text{-}Ves}$ HEC gel containing PI-*b*-PEG polymersomes with pH gradient, n.s.: not significant.

Supplementary references

[1] D. Benoit, V. Chaplinski, R. Braslau, C. J. Hawker, *J. Am. Chem. Soc.* **1999**, *121*, 3904.

[2] S. Matoori, Y. Bao, A. Schmidt, E. J. Fischer, R. Ochoa-Sanchez, M. Tremblay, M. Oliveira, C. F. Rose, J.-C. Leroux, *Small*, DOI 10.1002/smll.201902347.

[3] D. van Krevelen, K. te Nijenhuis, *Cohesive Properties and Solubility, in Properties of Polymers*, Elsevier, Amsterdam, Netherlands **2009**.
